# LNA-i-miR-221 activity in colorectal cancer: A reverse translational investigation

**DOI:** 10.1016/j.omtn.2024.102221

**Published:** 2024-05-20

**Authors:** Asad Ali, Katia Grillone, Serena Ascrizzi, Giulio Caridà, Lucia Fiorillo, Domenico Ciliberto, Nicoletta Staropoli, Pierosandro Tagliaferri, Pierfrancesco Tassone, Maria Teresa Di Martino

**Affiliations:** 1 Department of Experimental and Clinical Medicine, University Magna Græcia of Catanzaro, 88100 Catanzaro, Italy; 2 Phase 1 and Translational Oncology Unit, AOU Renato Dulbecco, Catanzaro, Italy; 3 Medical Oncology Unit, AOU Renato Dulbecco, Catanzaro, Italy

**Keywords:** MT: Oligonucleotides: Therapies and Applications, microRNAs, miRNA, miRNA inhibition, miR-221, LNA-i-miR-221, colorectal cancer, CRC, miRNA therapeutics, non-coding RNA therapeutics, RNA therapeutics

## Abstract

Colorectal cancer (CRC) is one of the most common malignancies and a relevant cause of cancer-related deaths worldwide. Dysregulation of microRNA (miRNA) expression has been associated with the development and progression of various cancers, including CRC. Among them, miR-221 emerged as an oncogenic driver, whose high expression is associated with poor patient prognosis. The present study was conceived to investigate the anti-CRC activity of miR-221 silencing based on early clinical data achieved from a first-in-human study by our group. Going back from bedside to bench, we demonstrated that LNA-i-miR-221 reduces cell viability, induces apoptosis *in vitro*, and impairs tumor growth in preclinical *in vivo* models of CRC. Importantly, we disclosed that miR-221 directly targets TP53BP2, which, together with TP53INP1, is known as a positive regulator of the TP53 apoptotic pathway. We found that (1) both these genes are overexpressed following miR-221 inhibition, (2) the strong anti-tumor activity of LNA-i-miR-221 was selectively observed on TP53 wild-type cells, and (3) this activity was reduced in the presence of the TP53-inhibitor Pifitrin-α. Our data pave the way to further investigations on TP53 functionality as a marker predictive of response to miR-221 silencing, which might be relevant for clinical applications.

## Graphical abstract

**Figure:**
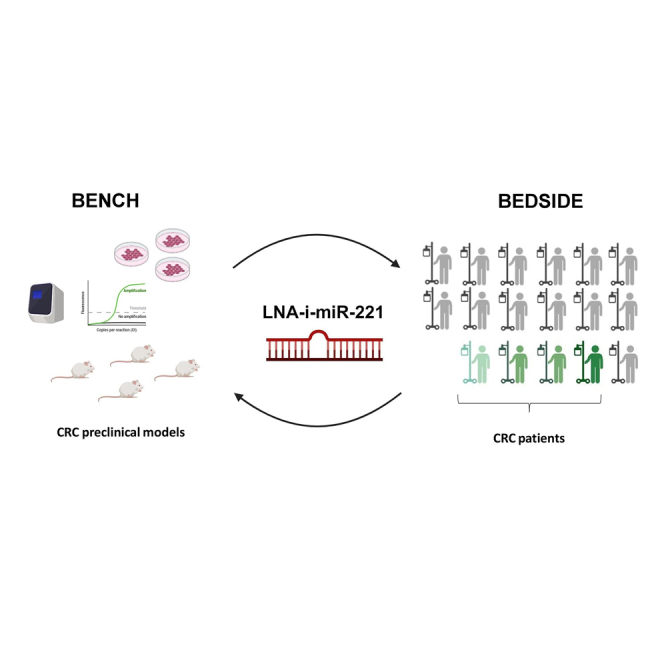

## Teaser

Di Martino and colleagues have previously demonstrated in a first-in-human trial that LNA-i-miR-221 exerts anti-tumor activity against colorectal cancer. Here, the authors investigated the molecular mechanisms that could underly this effect and observed hyper-activation of the TP53 pathway, due to overexpression of TP53BP2 and TP53INP1 miR-221 targeted genes.

## Introduction

Colorectal cancer (CRC) is one of the most common tumors worldwide with a high morbidity and mortality rate. Primary CRC can metastasize to distant organs like the peritoneum and liver, resulting in an incurable disease with a short survival rate.^1^ The median age of CRC at diagnosis is around 70 years and the risk is aging-related; however, the number of patients younger than 50 is increasing. Conventional CRC treatments include surgery, chemotherapy, radiotherapy, and molecular targeted therapies. However, despite the advancement in diagnostics and therapeutics, CRC remains an aggressive tumor with a poor clinical outcome.^2^^,^^3^^,^^4^^,^^5^ The design of innovative strategies and identification of new targets is, therefore, eagerly awaited. So far, several investigations focused on molecular events driving the multistep malignant CRC onset and progression.^6^^,^^7^^,^^8^ In this context, microRNAs (miRNAs) play a relevant role.^9^^,^^10^^,^^11^^,^^12^

miRNAs are small non-coding ∼22-nucleotides-long RNA sequences that bind in a partial complementary manner in the 3′ UTR region of mRNAs by exerting a strict control of gene expression via template degradation and translational repression.^13^ Since miRNAs modulate key biological pathways, including cell proliferation, cell differentiation, and apoptosis,^14^ their dysregulation contributes to cancer onset, progression, and response to therapy, depending on their oncogenic (oncomiR) or tumor suppressive functions.^15^^,^^16^ Among the most relevant tumor-associated miRNAs, we focused on the oncomiR-221, whose overexpression was found in several cancer types^17^ including thyroid cancer,^18^ breast cancer,^19^ hepatocellular carcinoma,^20^ multiple myeloma,^21^^,^^22^ glioblastoma,^23^ pancreatic cancer,^24^ and CRC.^25^ In particular, the high expression of miR-221 in CRC is adversely correlated to the patient’s survival.^14^ In terms of biological mechanisms, it has been reported that high expression of miR-221 in CRC facilitates tumor development through the inhibition of several genes including (1) QKI, by increasing cell growth,^26^ (2) TP53INP1, by inhibiting autophagy and enhancing cell proliferation,^27^ (3) p57/CDKN1C, by reducing apoptosis,^28^ and (4) SOCS-3, by promoting metastasis via the increase of angiogenesis process.^29^ Therefore, the role of miRNAs as biomarkers and potential anti-cancer targets has widely emerged.^30^^,^^31^

We have previously developed a 13-mer LNA inhibitor of miR-221 with a full phosphorothioate (PS)-modified backbone. This miRNA-221 inhibitor, named LNA-i-miR-221, efficiently downregulates miR-221, and then causes the upregulation of miR-221 canonical targets, and induces anti-tumor effects in several preclinical tumor models.^32^^,^^33^ It demonstrated favorable pharmacokinetic profiles in mice, rats, and monkeys with rapid and wide tissue distribution, without behavioral changes and/or organ-related toxicity, in GLP (Good Laboratory Practice) animal studies.^34^^,^^35^ These preclinical findings were translated in the context of a first-in-human, phase 1, dose-escalation study conducted on 17 patients with solid tumors completed in December 2021 (ClinicalTrials.gov: NCT04811898) aimed to evaluate safety and to identify the maximum tolerated dose and the recommended dose for phase 2 trials. This study highlighted an excellent safety profile of the treatment with the first-in-class LNA-i-miR-221 in all patients and achieved a major response in a CRC refractory patient.^36^ Moreover, three of four patients showing a trend of reduction in term of maximum tumor size of metastatic lesions had a CRC. Even if the limited number of patients with CRC included in the study requires a phase 2 trial on CRC patients to assess the tumor-specific sensitivity of miR-221 inhibition in this context, this early clinical evidence prompted us to turn back to the bench with the aim of exploring the biological and mechanistic effects of miR-221 inhibition on *in vitro* and *in vivo* preclinical models of CRC. Our findings shed light on this issue and warrant further bench and clinical investigation.

## Results

### Evaluation of LNA-i-miR-221 activity on *in vitro* models of CRC

**Figure 1:**
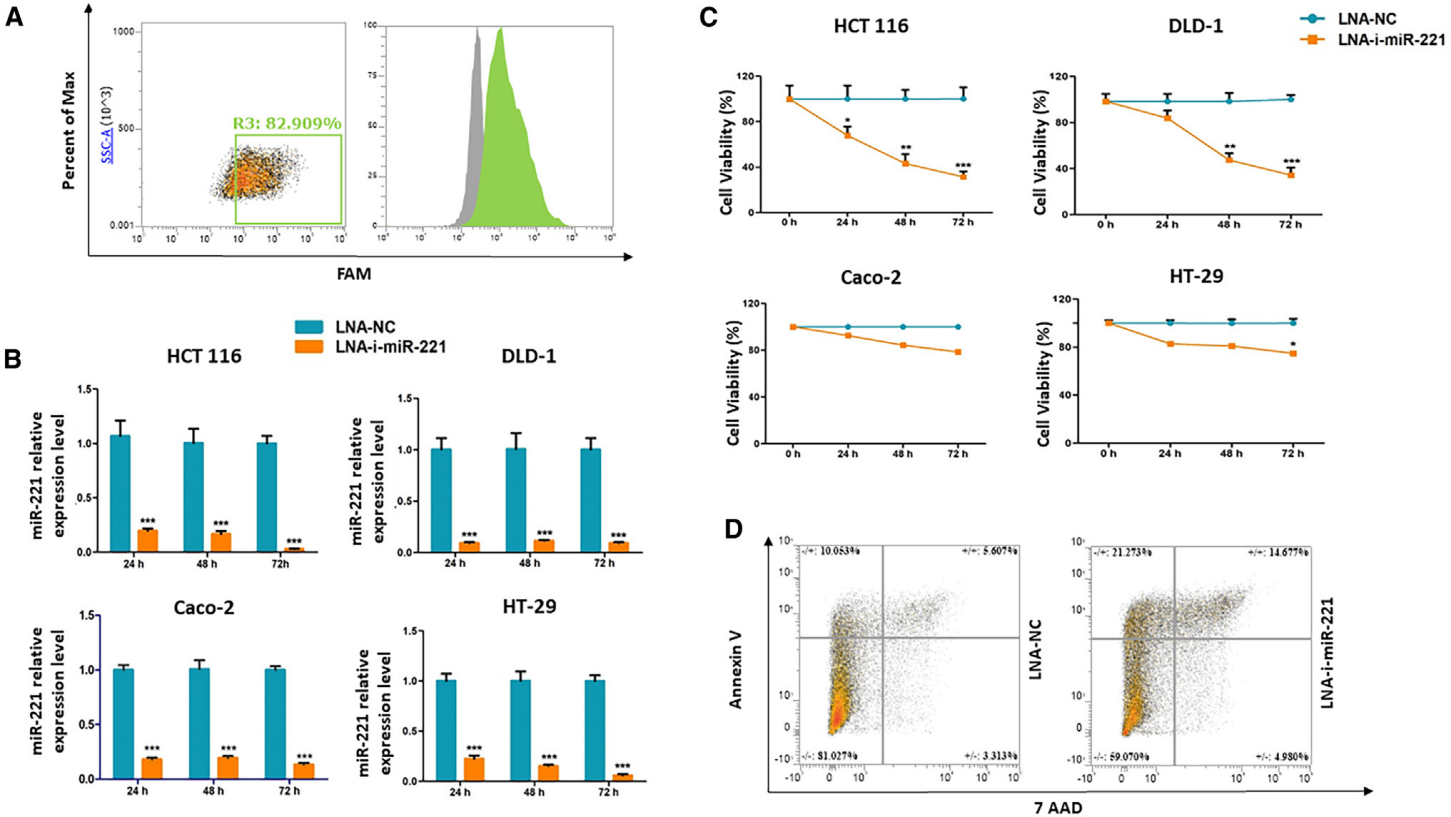
*In vitro* activity of LNA-i-miR-221 on CRC cell lines (A) percentage of FAM-labeled HCT 116 cells, 24 h following transfection with an FAM-LNA-NC. (B) Histogram bars representing the expression level of miR-221 at various time points after treatment with 15 nM of LNA-NC or LNA-i-miR-221. Values were normalized to the expression of RNU44. (C) Graphs representing the percentage of viable cells following treatment with 15 nM LNA-NC or LNA-i-miR-221. (D) Dot plots representing the distribution of HCT 116 cells stained with Annexin V/7AAD 24 h after treatment with 15 nM LNA-NC (left) or LNA-i-miR-221 (right). To calculate statical significance, Student’s t test was applied. ∗*p* < 0.05, ∗∗*p* < 0.01, ∗∗∗*p* < 0.001.

We evaluated the effect induced by treatment with LNA-i-miR-221 on HCT 116, Caco-2, HT-29, and DLD-1 cell lines as represented in *in vitro* models of CRC. To this aim, we adopted lipid-mediated transfection to deliver 15 nM of LNA-i-miR-221 or LNA-scramble, which served as negative control (LNA-NC), or a non-targeting LNA-FAM (locked nucleic acid-fluorescein acidities)-labeled oligonucleotide, that served as a positive control of transfection efficiency. Once a high LNA uptake (>80% of FAM positivity) via flow cytometry (Figure 1A) was confirmed, we checked the expression of miR-221 by quantitative real-time (qRT)-PCR at different time points after treatment with LNA-i-miR-221. We observed that miR-221 was significantly (∗∗∗*p* < 0.0001) downregulated in all treated cells as compared with cells transfected with LNA-NC as early as 24 h after transfection (Figure 1B). We then evaluated the effect on cell survival consequent to the target depletion 24 h, 48 h, and 72 h after treatment. We observed a significant time-dependent reduction in term of cell viability of HCT 116 and DLD-1 (decrease >60% within 72 h in both cell lines) and a moderate and less significant decrease of Caco-2 and HT-29 cell viability (Figure 1C). Since HCT 116 and DLD-1 cells showed similar response, we selected HCT 116 for further experiments. Annexin V/7AAD staining conducted on HCT 116 cells showed a 3-fold increase of late apoptotic markers (detected as cells positive for both Annexin V and 7AAD markers) after 24 h of transfection with LNA-i-miR-221, indicating induction of an apoptotic cell death (Figure 1D).

### Inhibition of miR-221 activates TP53 pathway in CRC

**Figure 2:**
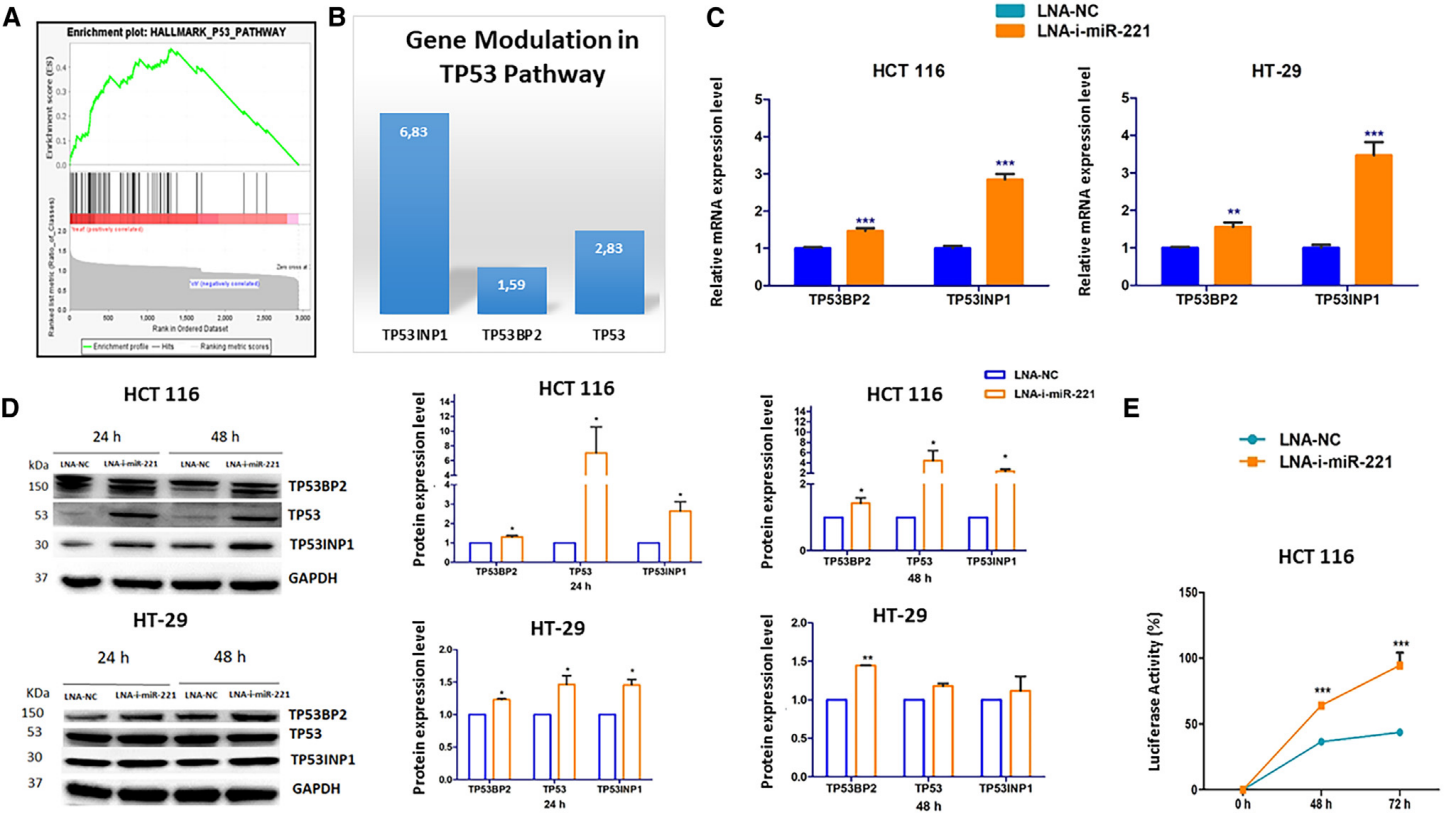
Molecular characterization of LNA-i-miR-221-mediated effect on CRC cells (A) GSEA highlighting a modulation of the TP53 pathway on HCT 116 cells after treatment with 15 nM LNA-i-miR-221 as compared with LNA-NC-treated cells (fold change [FC] ≤ −1.5 or ≥1.5 of differentially expressed genes was considered). (B) Histogram bars showing the FC of genes responsible for TP53 pathway modulation from GEP analysis. (C) Histogram bars representing mRNA expression of miR-221 targeted genes. GAPDH was used to normalize data. (D) Protein expression of miR-221-targeted genes, GAPDH was used to normalize data; densitometric analysis of bands resulting from the western blots 48 h following treatments at the indicated conditions is reported on the right. Values ± standard deviation (SD) represented on the x axis has been normalized on GAPDH for each experimental condition and then on values from LNA-NC. (E) Curves are representative of absorbance values that indicate luciferase activity; values were normalized to SEAP to obtain the represented percentage. To calculate statistical significance, Student’s t test was applied. ∗*p* < 0.05, ∗∗*p* < 0.01, ∗∗∗*p* < 0.001.

We then investigated, via gene expression profiling (GEP), the molecular mechanisms underlying the anti-tumor effect induced 24 h after exposure to LNA-i-miR-221 on HCT 116 cells. Gene set enrichment analysis (GSEA) highlighted the main pathways perturbed after miR-221 inhibition. Among them, the TP53 pathway showed the higher enrichment score (ES = −0.53). On the other hand, no genes annotated in the other pathways came up from the GSEA, with lower ES value, were identified in the list of genes selected by fold change analysis (FC = ±1.5). Figure 2A shows the enrichment plot of the highly activated TP53 pathway in cells transfected with LNA-i-miR-221 as compared with LNA-NC. To highlight the putative miR-221 targeted genes among those highly expressed in treated cells and involved in the modulation of this pathway, we adopted Target Scan and mirDB tools and we identified TP53BP2 and TP53INP1, which are pre- and post-transcriptional activators of the TP53 pathway, respectively. The higher expression of both these genes in treated cells, highlighted by microarray analysis (Figure 2B), was also validated via qRT-PCR at the mRNA level (Figure 2C) and by western blotting (WB) at the protein level (Figure 2D) on HCT 116 and HT-29. We observed that both targets were positively modulated after treatment with LNA-i-miR-221 on both cell lines. Since only TP53INP1 has been previously described as an miR-221 direct target,^27^ we investigated the direct binding of miR-221 to the 3′UTR region of TP53BP2, via dual luciferase assay (Figure 2E). The luciferase vector encoding for 3′UTR TP53BP2, Gluc (Gaussia luciferase), and SEAP (secreted alkaline phosphatase) reporters was co-transfected in HCT 116 cells together with LNA-i-miR-221 or LNA-NC. The luciferase activity increased in cells treated with the miR-221 inhibitor confirming the notion that the expression of TP53BP2 is inversely associated with expression of miR-221. We then demonstrated that TP53BP2 is a direct target of miR-221.

**Figure 3:**
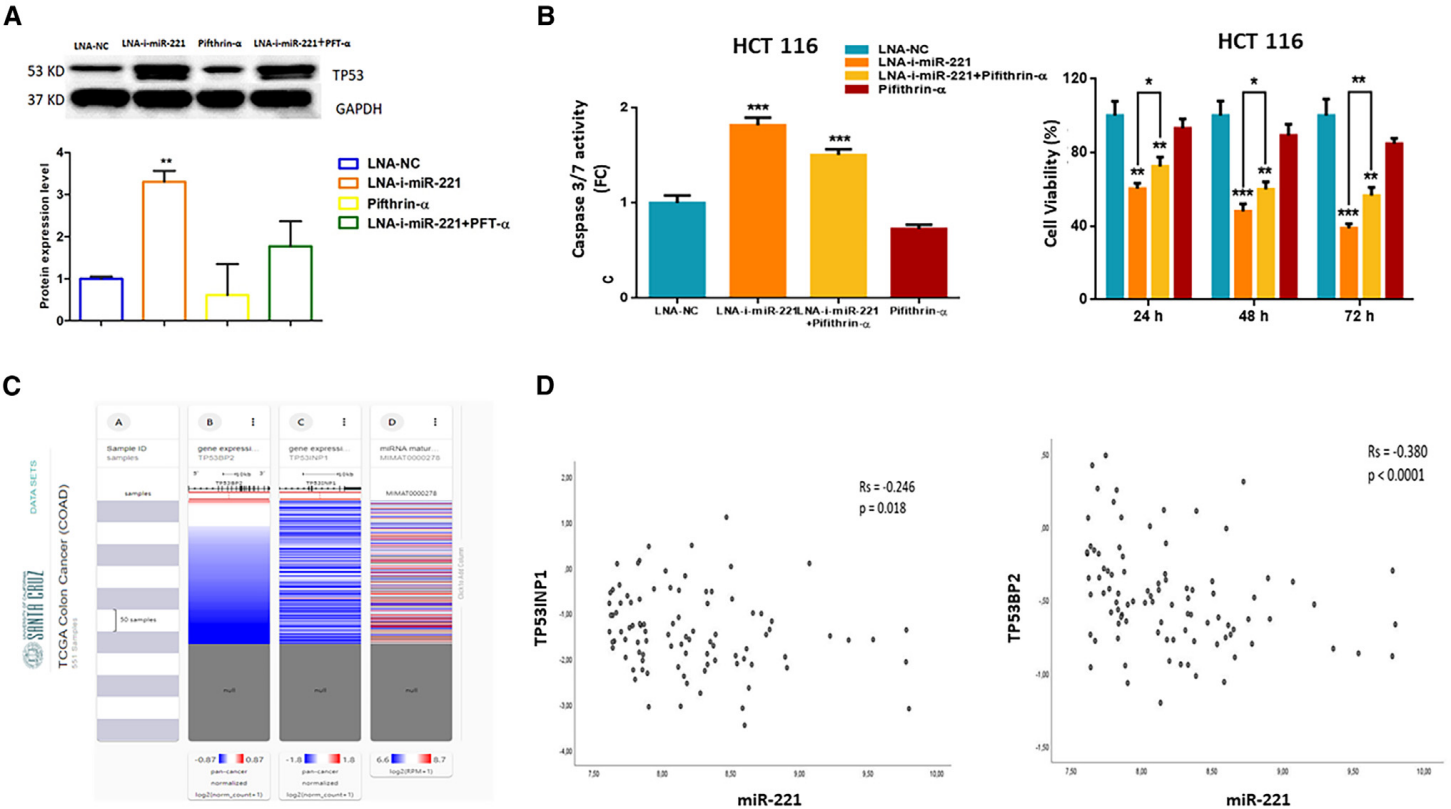
LNA-i-miR-221 dependent activation of the p53 pathway (A) Expression of TP53 protein after treatment with 15 nM of LNA-NC or LNA-i-miR-221. GAPDH was used to normalize data. Histogram bars in the lower panel are representative of densitometric analysis of bands resulting from the western blots, after normalization on GAPDH and LNA-NC. (B) Fold change values indicating the activity of caspase 3/7 assessed (left); histogram bars showing the percentage of viable cells for each experimental condition at the indicated time points (right). All represented values have been normalized on LNA-NC. To calculate statical significance, Student’s t test was applied. ∗*p* < 0.05, ∗∗*p* < 0.01, ∗∗∗*p* < 0.001. (C) UCSC Xena browser visual spreadsheet in the COAD dataset. “Null” identifies patients for whom the gene expression has not been collected. Each column identifies an RNA as described on the top. Expression is colored blue to red for low and high expression, respectively. (D) Scatterplot for Spearman correlation. On the x axis miR-221 expression, or the y axis TP53INP1 (left) and TP53BP2 (right) expression.

To further validate the hypothesis of a direct modulation of the TP53 pathway mediated by miR-221 inhibition, we took advantage of the cell-permeable chemical inhibitor of TP53 named Pifithrin-α. At this aim, we treated HCT 116 cells with Pifithrin-α 1 h before transfection with LNA-i-miR-221 or LNA-NC or vehicle. Twenty-four hours later, we evaluated the consequent changes induced on (1) the TP53 protein level, (2) the onset of an apoptotic pathway by assessing the activity of caspase 3 and 7, and on (3) cell viability. We observed that TP53 expression (Figure 3A) and caspase 3/7 activity (Figure 3B left panel) was reduced, while cell viability was increased (Figure 3B right panel) in cells treated with both LNA-i-miR-221 and Pifitrin-α, as compared with cells treated with LNA-i-miR-221 alone. Our findings highlighted that the anti-tumor activity induced by treatment with LNA-i-miR-221 take advantage from the activation of the TP53 pathway, which is probably led by the already known TP53INP1 and the newly discovered TP53BP2 miR-221 target genes.

To validate the anti-correlation between miR-221 and TP53BP2 expression on a patient’s dataset, we utilized the Xena browser to evaluate the expression correlation of miR-221, TP53BP2, and TP53INP1 in a dataset including 186 patients, as shown in Figure 3C. We split patients into the miR-221 high expression group and miR-221 low expression group, according to the miR-221 median level in the tumor tissues. In the miR-221 high expression group, there was a negative correlation between miR-221 and TP53INP1 (Rs = −0.246, *p* = 0.018) (Figure 3D left panel) and between miR-221 and TP53BP2 (Rs = −0.380, *p* = 0.0001) (Figure 3D right panel). In the miR-221 low expression group, there was a negative correlation between miR-221 and TP53INP1 only (Rs = −0.290, *p* = 0.005), while no correlation was found in the miR-221 low expression group between miR-221 and TP53BP2 (Rs = 0.064, *p* = 0.541) (data not shown).

### LNA-i-miR-221 is effective on *in vivo* models of CRC

**Figure 4:**
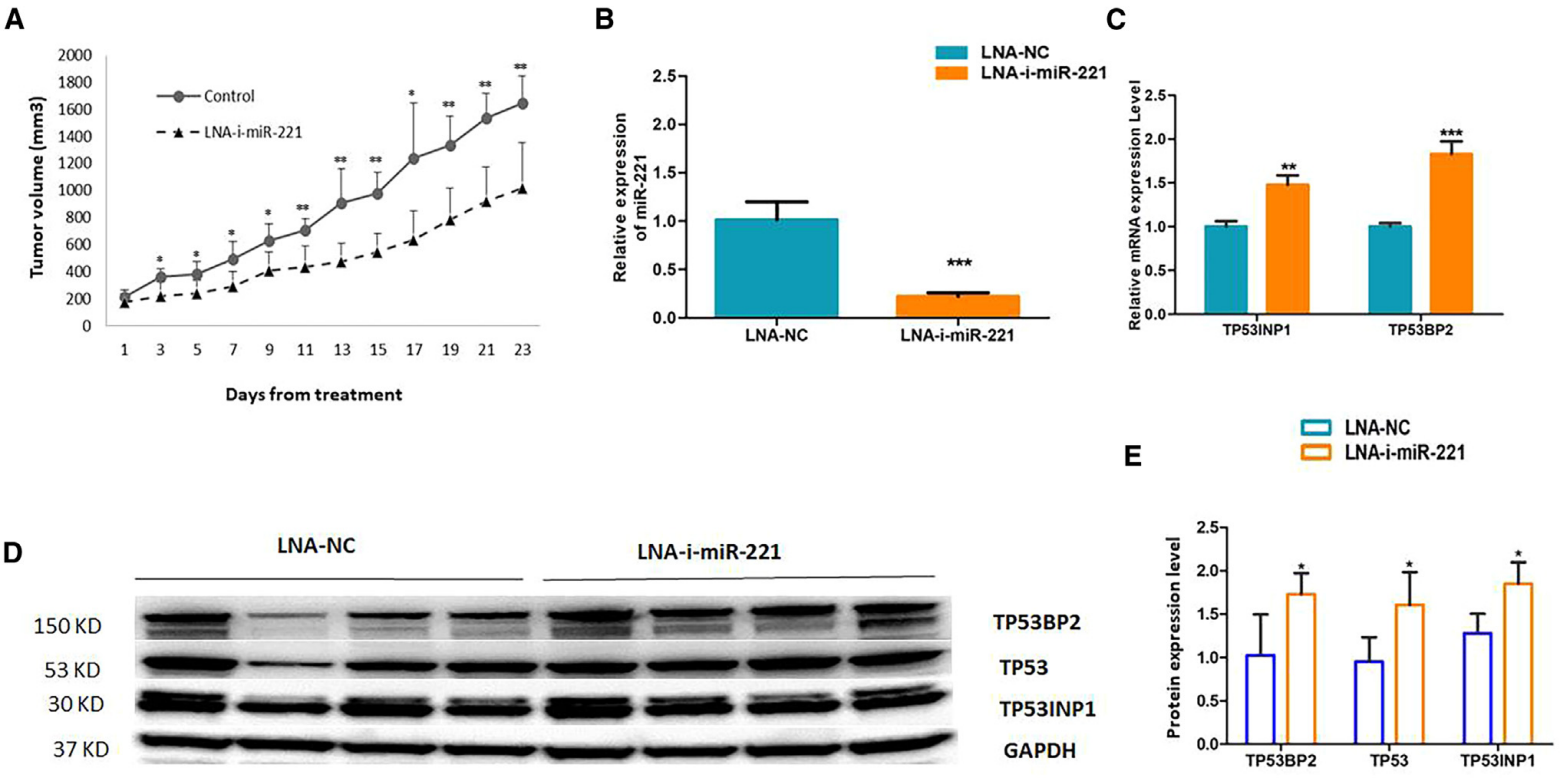
*In vivo* activity of LNA-i-miR-221 in CRC xenograft models (A) The graph represents the tumor volume ± standard deviation (SD) on CRC xenograft models treated with LNA-i-miR-221 or LNA-NC. (B and C) Histogram bars represent expression levels of miR-221 (B) and miR-221 target genes (C) evaluated on tumors retrieved from treated mice and controls; RNU44 and GAPDH were used to normalize qRT-PCR miRNA and mRNA data, respectively. (D) Protein expression of miR-221 target genes assessed on protein extracted from retrieved tumors. (E) Densitometric analysis of western blot signals was performed by normalizing each band on GAPDH and on LNA-NC. Resulting data from independent replicates have been reported on the x axis of the histogram bars. Student’s t test was applied. ∗*p* < 0.05, ∗∗*p* < 0.01, ∗∗∗*p* < 0.001.

Finally, to investigate the activity of LNA-i-miR-221 in animals, we generated HCT 116 xenograft models on NOD-SCID mice. Mice were randomized into two groups and were treated with LNA-i-miR-221 or LNA-NC (25 mg/kg) via intraperitoneal injection from Monday to Friday. After 3 weeks, tumor volumes of LNA-i-miR-221-treated mice were significantly reduced as compared with the control group (Figure 4A). Engrafted tumors were then retrieved to explore the molecular bases underlying the anti-tumoral activity of LNA-i-miR-221 in CRC tumors. In agreement with our *in vitro* findings, we observed through qRT-PCR a strong down-modulation of miR-221 (Figure 4B) and an increased expression of mRNA (Figure 4C) and protein (Figure 4D) level of TP53BP2 and TP53INP1 genes, in treated mice compared with controls.

## Discussion

MiR-221 is one of the most extensively studied oncomiRs due to its pivotal role in the progression of several cancer types, in which a dysregulated high expression has been reported.^17^^,^^37^ Among them, CRC is the second leading cause of tumor-related death worldwide.^38^ In these patients, high expression of miR-221 is strongly associated with short survival^14^^,^^39^ and increase of tumor cell proliferation, invasion, and metastatic spread.^28^^,^^40^ It is not surprising that the targeting of miR-221 as an innovative therapeutic approach for CRCs moved into the spotlight for medical research.^41^

Our group recently reported early clinical data on the safety and activity of the first-in-class LNA miR-221, a selective miR-221 inhibitor, which has been demonstrated to be an active agent in refractory advanced cancer patients. Although the primary endpoint was the identification of the recommended dose for the phase 2 trial, explorative studies about the clinical response have been performed. In particular, one 60-year-old patient with a metastatic refractory CRC exhibited a major response upon treatment with 5 mg/kg of LNA-i-miR-221. Moreover, among 17 patients included in the study cohort, three out of four showing a reduction, in terms of tumor size, were CRC patients.^36^ This clinical evidence rekindled our interest to explore the molecular mechanisms underlying the observed anti-CRC activity of LNA-i-miR-221, on *in vitro* and *in vivo* preclinical models. In this regard, here we confirmed that the treatment with LNA-i-miR-221 is effective in reducing proliferation and inducing apoptosis in CRC cell lines, and to impair tumor growth in CRC mouse xenograft models. Moreover, we explored the transcriptomic changes following LNA-i-miR-221 exposure with the aim to shed light on the perturbed pathways and to disclose novel potential miR-221 targets. Previous studies reported that TP53INP1,^27^ SPINT1,^42^ SOCS-3,^29^ RECK,^40^ and QKI^26^ are direct targets of miR-221 able to contribute to CRC progression. On the other hand, the expression of TP53BP2 has been reported to be downregulated in CRC^43^ and also in other tumors where the apoptotic process is inhibited, and cell migration is favored by cell polarity disruption.^44^ Among the pathways perturbed following miR-221 inhibition in our *in vitro* models, we focused on TP53, playing a crucial role in CRC tumorigenesis,^45^^,^^46^ which emerged as the pathway with the highest enrichment score.

The hyper-activation of the TP53 network after treatment with LNA-i-miR-221 is probably due to the overexpression of TP53INP1 and TP53BP2 genes following miR-221 inhibition. Proteins encoded by these two genes are able to bind TP53 and activate TP53-dependent apoptotic cascade.^47^^,^^48^ TP53INP1 has been already described as an miR-221 direct target and it activates TP53 at the post-transcriptional level and stabilizes the TP53 expression to start downstream pathways.^27^^,^^49^ To the best of our knowledge, we reported for the first time and validated via luciferase assay the evidence for TP53BP2 direct miR-221 targeting, confirmed by *in vitro* and *in vivo* CRC models, as well as supporting their lower expression in patients highly expressing miR-221.

TP53 is widely considered an important barrier against the carcinogenic process because it responds to various stress signals in cells and converts them into different outcomes including cell-cycle arrest or cell death or activation of DNA repair mechanisms.^50^^,^^51^ In human cancer, the TP53 gene is inactive either because of loss-of-function mutations or by negative regulation of wild-type protein product.^52^ Inactivation of TP53 in CRC is caused by mutations in 43% of tumors, while in other cases its function is antagonized by other interacting proteins.^53^

Our hypothesis concerning the possible relationship between LNA-i-miR-221 activity against CRC and activation of the TP53 pathway was supported by the observation that TP53 wild-type cells (HCT 116 and DLD-1) were sensitive to LNA-i-miR-221, while TP53 mutated cells (HT-29 and Caco-2) showed non-significant response. Although the target genes TP53BP2 and TP53INP1 were positively modulated also in HT-29 after treatment with LNA-i-miR-221, the increase was not accompanied by an apoptotic and strong anti-proliferative effect, probably due to TP53 loss-of-function mutations, which impair the functionality of the TP53 pathway on this cell line. The combinatory treatment of HCT 116 cells with the miR-221 inhibitor and Pifithrin-α, which is able to block TP53 expression at the mRNA level,^54^ demonstrated that, in the presence of this compound, the effectiveness of LNA-i-miR-221 is reduced. Even if Pifithrin-α did not strongly antagonize LNA-i-miR-221 activity, this result further confirms that the anti-tumor activity of LNA-i-miR-221 in CRC may, at least in part, be improved by TP53 activation.

We provided a proof-of-concept study from bench to bedside and back, which paved the way to the investigation between miR-221 and TP53BP2 expression in large pan-cancer datasets to disclose the same target regulation in other cancer types. Unfortunately, we were not able to validate the TP53 mutational status, and the expression of miR-221, and miR-221-targeted genes, on tumor deposits of the CRC patients included in the clinical study published by Tassone et al.^36^ However, we aim to validate the proposed mechanism in a larger cohort of patients subjected to LNA-i-miR-221 treatment, for instance in a phase 1B/2 trial, to explore, as the secondary endpoint, biomarker assessment including TP53 mutational status, expression of TP53 upstream or downstream regulators, and miR-221 targets, by the use of tumors or liquid biopsy for circulating-nucleic acids evaluation. The relevance of this study relies on the prospective use of a selection criterion to stratify CRC patients who may benefit from the treatment with LNA-i-miR-221.

## Materials and methods

### Cell lines

HCT 116, DLD-1, Caco-2, and HT-29 cell lines were kindly provided by the Department of Experimental and Clinical Medicine of the University Magna Græcia of Catanzaro. Among the available CRC cell lines, we selected those whose high expression of miR-221 has been reported in the literature.^25^ HCT 116, Caco-2, and HT-29 cells were cultured in DMEM supplemented with 10% fetal bovine serum (FBS) and 1% penicillin/streptomycin (P/S), while DLD-1 was cultured in RPMI medium (10% FBS and 1% P/S). These cell lines were maintained at 37°C/5 CO_2_ and assessed as mycoplasma-free prior to use.

### Oligonucleotide synthesis

The LNA-i-miR-221 and LNA-scramble (that served as the negative control) oligonucleotides were purchased by Exiqon (Vedbaek, Denmark) as described previously. LNA-i-miR-221 is able to selectively target miR-221, whose sequence is conserved among species, including mice.^32^^,^^55^^,^^56^

### Transfection and drug treatment

Fifteen nanomoles of LNA-i-miR-221 or LNA-scramble were delivered in HCT 116, DLD-1, Caco-2, and HT-29 cell lines by using Lipofectamine 3000 according to the manufacturer’s instructions. Cyclic Pifithrin-α hydrobromide (Abcam, Cambridge, UK) was added to HCT 116 at a final concentration of 10 μM, 1 h prior to the transfection conducted as mentioned above.

### Cell proliferation and apoptosis assays

Cell proliferation rate and apoptosis assays were conducted as previously reported.^57^ Briefly, cell viability and caspase 3/7 activity were evaluated at different time points after transfection and/or drug treatment by Cell Titer Glo and Caspase-Glo luminescent assays, respectively (Promega, Madison, WI, USA) according to standard protocols. Glomax multi-detection system (Promega, Madison, WI, USA) was used to record luminesce values. Apoptosis was assessed 24 h after transfection via Annexin V/7AAD staining following standard procedures (BD, Franklin Lakes, NJ, USA) by flow cytometry on Attune NtX instrument (Thermo Fisher Scientific, Waltham, MA, USA).

### RNA extraction, reverse transcription, and quantitative real-time PCR

Total RNA from collected cells was extracted by using TRIzol reagent (Gibco, Life Technologies, Carlsbad, CA), according to the manufacturer’s instructions. RNA quantification was performed by a NanoDrop ND 1000 spectrophotometer (Thermo Fisher Scientific, Waltham, MA, USA). To evaluate the expression of miR-221, qRT-PCR was performed as previously reported.^22^ Briefly, 15 ng of RNA was used for reverse transcription by Taqman microarray kit (Thermo Fisher Scientific, Waltham, MA, USA). qRT-PCR was performed by using Taqman Fast universal PCR master mix (Thermo Fisher Scientific, Waltham, MA, USA) by using Taqman probe assay ID 000524 to evaluate miR-221 expression and Taqman probe assay ID 001094 to evaluate RNU44 expression that was used to normalize data.

To evaluate the expression of TP53INP1 and TP53BP2, 500 ng of RNA was used for reverse transcription performed with a High Capacity cDNA Reverse Transcription Kit (Applied Biosystem, Thermo Fisher Scientific, Waltham, MA, USA). qRT-PCR was then performed by SYBR Green PCR Master Mix (Thermo Fisher Scientific, Waltham, MA, USA) to detect TP53INP1 and TP53BP2 expression level by using primers listed in Table 1, GAPDH was used as the internal control to normalize expression data.

**Table 1:** Sequence of primers used in the study

Primer ID	Sequence
TP53INP1 Forward	5′- TCTTCCTCCAACCAAGAACCA-3′
TP53INP1 Reverse	5′-TGAAGGGTGCTCAGTAGGTG-3′
TP53BP2 Forward	5′-AGCTTGATCGCCTCTATAAGGA-3′
TP53BP2 Reverse	5′-CCCTCAGCTCATTAACACGCT-3′
GAPDH Forward	5′-GAG TCA ACG GAT TTG GTC GT-3′
GAPDH Reverse	5′- GAC AAG CTT CCC GTT CTC AG-3′

All qRT-PCRs were performed on a VIIA7 qRT-PCR instrument (Life Technologies by Thermo Fisher Scientific, Waltham, MA, USA).

### Gene expression profiling

Total RNA was extracted by the TRIzol method (Gibco, Life Technologies, Carlsbad, CA) from HCT 116 cells, 24 h after transfection with 15 nM LNA-i-miR-221 and LNA-NC, performed in two independent biological duplicates. One hundred nanograms of each RNA was expedited through the Gene Chip WT PLUS Reagent Kit (Thermo Fisher Scientific, Waltham, MA, USA) according to the manufacturer’s instructions and then the hybridization by Gene Chip Human Transcriptome 2.0 was performed (Thermo Fisher Scientific, Waltham, MA, USA). The GeneChip TM 3000 system was used to proceed the hybridization, staining, and washing process. Resulting data (CEL files) were produced by Gene Chip Command Console software and further analyzed by Transcriptome Analysis Console (TAC) software v 4.0 (Applied Biosystems, Thermo Fisher, Waltham, MA, USA). Data were normalized by the Signal Space Transformation-Robust Multi-Array Average (SST-RMA) algorithm. Difference of gene expression after inhibition of miR-221 was identified by the fold change (FC) ≤ −1.5 or ≥1.5 and *p* value <0.05. HTA 2.0 library available on TAC 4.0 software was used to annotate results. The differentially expressed genes were analyzed by Gene Set Enrichment Analysis 4.3.2 (GSEA) software.^57^

### Bioinformatics analysis

Target scan^58^ and miRDB^59^ tools were used to predict the putative miR-221-targeted genes.

### Luciferase assay

To validate the direct binding of miR-221 to the 3′UTR region of TP53BP2, we adopted the well-established method of the Luciferase assay.^60^^,^^61^^,^^62^^,^^63^ For this purpose we adopted the Secret-Pair TM Dual Luminescence Assay Kit (GeneCopoeia), which consists of a dual-reporter system cloned in an all-in-one vector allowing data normalization, by eliminating the impact of transfection efficiency. One microgram of the pEZX-MT05 vector encoding the 3′UTR region for TP53BP2, Gluc (Gaussia luciferase), and SEAP reporters (catalog No# HmiT018178-MT05), purchased from GeneCopoeia, was co-transfected with 15 nM LNA-i-miR-221 or LNA-NC and then luciferase activity was checked, according to the manufacturer’s protocol, 48 and 72 h later. SEAP was used as a normalizer of luciferase activity.

### Western blotting

Whole protein extracts were recovered from cells by NP-40 lysis buffer containing the halt protease and phosphatase inhibitor cocktail (Thermo Fisher Scientific, USA). Western blot analysis was conducted as described previously.^16^ The protein expression was checked by using the following primary antibodies: TP53INP1 (ab202026) and TP53BP2 (ab181377) from Abcam (Cambridge, UK), TP53 (2527S) from Cell Signaling Technology (Massachusetts, USA), and GAPDH (SC25778) from Santa Cruz Biotechnology (Dallas, TX, USA). The secondary anti-rabbit IgG HRP-linked antibody (#7074) was purchased from Cell Signaling Technology (Danvers, MA, USA).

### Patient dataset analysis

In order to verify the correlation between miR-221 expression and TP53BP2 and TP53INP1 levels in the patient dataset, the Xena platform from the University of California Santa Cruz (UCSC) was used to extract RNA expression levels.^64^^,^^65^ Through the Xena browser, single gene per single patient RNA expression from The Cancer Genome Atlas (TCGA) was gathered from the Colon Adenocarcinoma (COAD) dataset. TP53BP2 and TP53INP1 RNA levels were extracted by the “IlluminaHiSeq_RNASeqV2” platform, and unit of measurement per single patient was defined as pan-cancer normalized log2(norm_count+1). For miR-221 levels, the “IlluminaHiSeq_miRNASeq” platform was used, and unit of measurement per single patient was defined as log2(RPM+1). Patients extracted were divided into “miR-221 high expression” and “miR-221 low expression” according to miR-221 median levels. Due different units of measurement and non-normal data distribution in the Shapiro-Wilk test, the Spearman test was performed to explore the correlation among miR-221, TP53BP2, and TP53INP1 for all statistical analysis (IBM SPSS Statistics for Windows, Version 28.0 Released 2021, Armonk, NY: IBM Corp). The strength of a correlation was defined according to R square value (positive or negative).^66^

### Animal and *in vivo* model of CRC

All standard guidelines and protocols recommended by national and institutional ethical committees were adopted for *in vivo* studies (Aut.Min.Sal n. 1138/2020-PR); 5 × 10^6^ HCT 116 cells were subcutaneously injected on the right side of each of 10 CB/17/non-obese diabetic-severe combined immunodeficiency (NOD-SCID) 6- to 8-week-old mice (Harlan Laboratories, lnc., Indianopolis, IN, USA). Ten days after injection, tumor masses were palpable. Mice were then randomly divided into two groups (five mice per group) and daily treated with 25 mg/kg of LNA-i-miR-221 or LNA-scramble via intraperitoneal injection as previously described.^67^^,^^68^ Tumor volume was assessed every 2 days via digital caliper measurements. When the size of the tumor reached 2 cm in diameter, the animals were euthanized and tumors were retrieved and stored in RNA Later for subsequent RNA extraction or at −80°C for subsequent WB analysis.

### Statistical analysis

Data were analyzed by GraphPad Prism version 5. Student’s t test was applied to calculate statistics and a *p* < 0.05 value was considered as cutoff of significance.

## Data and code availability

Data can be provided on reasonable request.
